# Fetal ocular development in the second trimester of pregnancy documented by 7.0 T postmortem Magnetic Resonance Imaging

**DOI:** 10.1371/journal.pone.0214939

**Published:** 2019-04-04

**Authors:** Zhonghe Zhang, Xiangtao Lin, Qiaowen Yu, Gaojun Teng, Fengchao Zang, Ximing Wang, Shuwei Liu, Zhongyu Hou

**Affiliations:** 1 Department of Medical Imaging, Shandong Provincial Hospital Affiliated to Shandong University, Shandong, China; 2 Department of Medical Imaging, The First Affiliated Hospital of Shandong First Medical University, Shandong, China; 3 Research Center for Sectional and Imaging Anatomy, Shandong University School of Medicine, Shandong, China; 4 Department of Radiology, Zhong Da Hospital, Southeast University School of Clinical Medicine, Jiangsu, China; University of Pennsylvania Perelman School of Medicine, UNITED STATES

## Abstract

Few investigators have analyzed fetal ocular growth with Magnetic Resonance Imaging (MRI) of high magnetic strength. Our purpose is to obtain normative biometrics for fetal ocular development in the second trimester of pregnancy. Sixty specimens with a gestational age (GA) of 12–23 weeks were scanned using a 7.0 T MRI scanner. The linear interocular and binocular distances (IOD and BOD, respectively), globe diameter (GD) and lens diameter (LD) were measured on the transverse section of the largest diameter of the eyeballs. The three dimensional (3D) visualization model of the eyeball was reconstructed with Amira software. Then, the globe and lens volumes (GV and LV, respectively) were obtained. All the measurements were plotted as a function of GA. The fetal ocular structures in the second trimester of pregnancy could be clearly delineated on 7.0 T postmortem MRI images. All the linear measurements logarithmically increased with GA, while, the volumetric measurements linearly increased with GA. Postmortem MRI of high magnetic strength can clearly document fetal ocular growth in the second trimester of pregnancy. These quantitative data may be a valuable reference for the assessment of normal fetal eyeball development in clinical settings and may be considered a supplement to anatomical investigations.

## Introduction

The morphology of each organ changes gradually during the normal fetal developmental process in vivo. Biometric data are an important diagnostic reference in the assessment of fetal health for all imaging examinations, and large datasets from previous research can be found in the literature [1–6]. Although eyeball and orbital imaging examinations are not routinely performed in obstetrical ultrasound (US) during the early stages of pregnancy, it is a reasonable and essential expectation to perform them for a detailed anatomical scan [7, 8]. A previous study discovered that quite obvious ocular pathologies have been missed on US in clinical settings, particularly if they are bilateral and symmetrical [8]. Additionally, the diagnoses are not convincing and may even be completely altered if a coexisting ocular pathology is discovered [8]. Therefore, assessment of fetal ocular development has been routinely included in fetal Magnetic Resonance Imaging (MRI) or US examinations, and it is important to obtain precise quantitative measurements during the normal growth process.

The discovery of abnormal eyeball and orbital dimensions may be associated with evidence of fetal dysgenesis or suspected central nervous system (CNS) malformation [8–11]. The developmental process of the fetus in vivo can be divided into three periods. They are the first, second, and third trimesters, which are closely correlated with the different developmental stages of the CNS. The second trimester is a period of very active neurogenesis and neuronal migration and plays an important role during in vivo life. Many congenital diseases are more likely to arise in the second trimester, and this period may be the earliest phase during which congenital diseases can be first detected and diagnosed in clinical imaging examinations [2, 3, 6].

Most of the in vivo fetal MRI scans are performed during the second half of gestation. This is partly due to the inadequate signal intensities produced by the small and moving fetal organs in early pregnancy. In vivo fetal MRI resolution is limited by partial volume averaging, decreased imaging time and increased slice thickness of the acquisition, making it difficult to obtain images of high resolution during the first and early second trimesters [12]. At present, the fetal eyeball is mostly studied in the second half of gestation with US and in vivo MRI [8–14] and mainly focuses on linear measurements without three dimensional (3D) reconstruction of the eyeball [12]. However, few investigators have delineated the ocular structures with MRI of high magnetic strength. Therefore, we still lack reliable and consistent, quantitative 3D measurements of the fetal eyeball in the early developmental phase, which would help to complete the record of normal development throughout gestation [12].

## Materials and methods

### Subjects

A total of 95 fetal specimens of 12–23 weeks gestational age (GA) were collected. They were from medically indicated or spontaneous abortions and fetal deaths from hospitals in Shandong Province, China. The subjects included in this research were partially or totally used to study fetal adrenal gland and brain development characteristics [1–3], laminar organization [4], cortical folding [5] and fetal brain templates [6] in our previous publications.

First, the chosen specimens had to have an anatomically normal and developmentally appropriate fetal brain, and they had to meet the following inclusion criteria, which were the same as in our previous research [1–6]: 1) maternal pregnancy records without indication of stressful intrauterine conditions, history of maternally transmitted genetic disease in the family, documented fetal chromosomal abnormalities, or a history of seizures in the case of eclampsia; 2) all the examinations indicating an anatomically normal and developmentally appropriate fetal CNS, based on the results of US examination for the fetus during pregnancy and results of postmortem MRI examinations of the specimen; and 3) no detectable CNS malformations from further validated, detailed autopsy combined with neuropathologic examinations.

Second, the eyeball and the adjacent structures of the selected specimens were not deformed and could be clearly discriminated on the MRI.

Sixty specimens were finally selected for the study. Their race was all Chinese Han. The GA distribution, number and sex of the chosen specimens are listed in Table 1.

**Table 1:** GA distribution, number and sex of chosen specimens (n = 60).

GA	Number	Sex (female/male)	GA	Number	Sex (female/male)
12	3	2/1	18	5	2/3
13	3	1/2	19	5	1/4
14	2	2/0	20	11	6/5
15	4	3/1	21	10	7/3
16	4	0/4	22	4	2/2
17	7	2/5	23	2	0/2

The length of the postmortem time (the time interval between delivery and collection of specimen) for each specimen was different and was as short as possible; it ranged from several minutes to a few hours. Once they were obtained, the specimens were immersed in 10% formalin for preservation with the anterior fontanelle slit open. They were preserved separately in wide-mouthed bottles made according to their body size to avoid deformation during storage and transportation; then, they were scanned with a 7.0 T MRI scanner as soon as possible. The time interval between the collection of specimens and the scanning was less than 2 months. This study was conducted with the approval of the Ethical Committee at the School of Medicine, Shandong University (Permit Number: 2012033). The parental written consent to donate the fetal cadaver was obtained.

### Image acquisition

A 7.0 T micro-MRI scanner with a maximal gradient of 360 mT (70/16 PharmaScan; Bruker BioSpin, Bremen, Germany) was used. We selected a rat body coil with an inner diameter of 60 mm to scan the fetal head. The scanning parameters were as follows. For T1-weighted images, the section thickness was 0.8 mm; section interval, 0.8 mm; TR, 384.4 ms; TE, 15.8 ms; matrix size, 512× 512; number of excitations, 1; field of view, 6×6 cm. For T2-weighted images, the section thickness was 0.5 mm; section interval, 0.5 mm; TR, 17,000 ms; TE, 50 ms; matrix size, 256×256; number of excitations, 4; and field of view, 6×6 cm.

### 3D reconstruction and quantitative measurements

**Fig 1:**
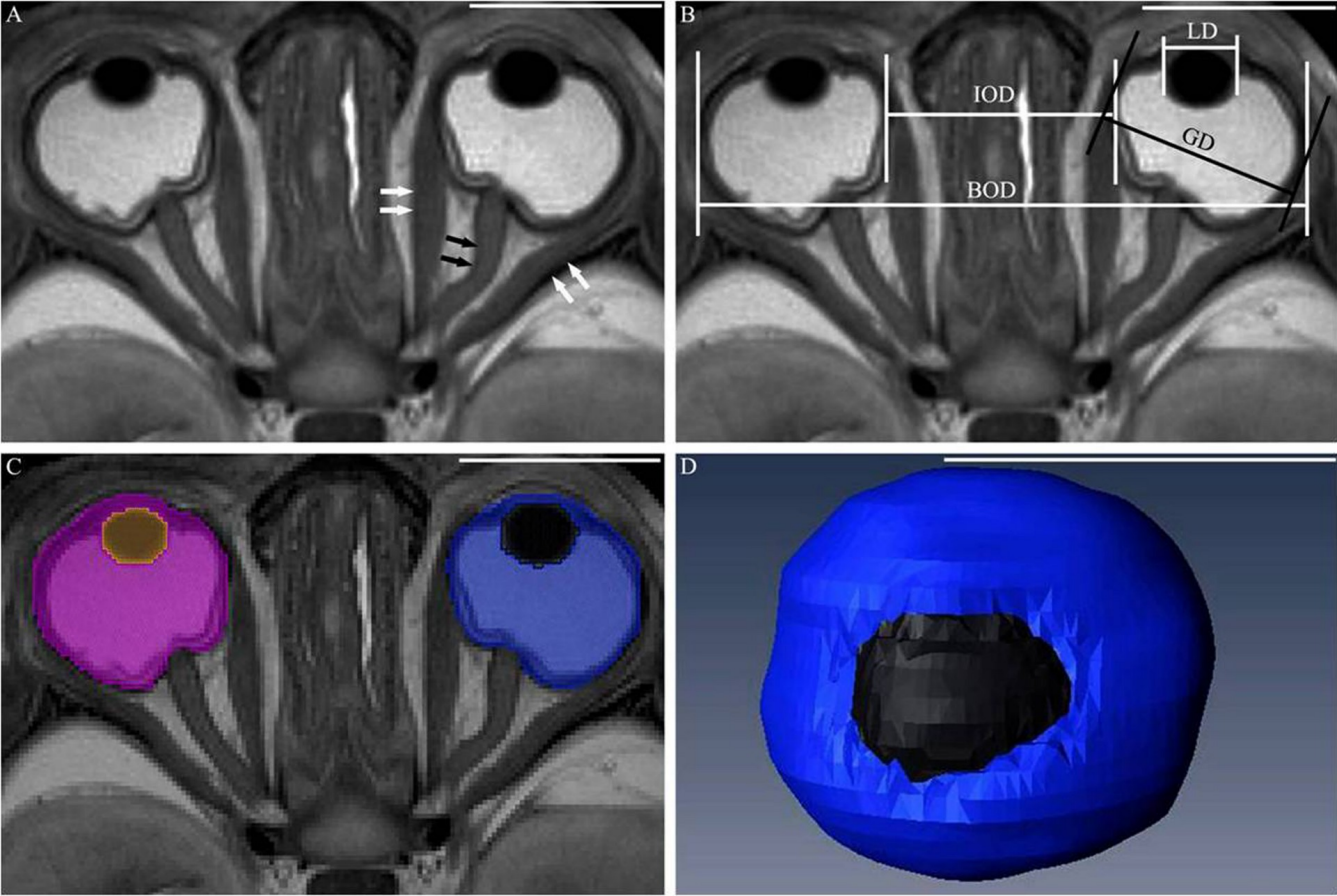
**Transverse T2-weighted 7.0 T MRI scans of the fetal ocular structures of 20 weeks GA (A) and their linear measurements (B), manual segmentation (C) and a 3D visualization model of the eyeball (D)**. The lens, vitreum, three layers of the eyeball wall, optic nerve (black arrows) and ocular muscles (white arrows) can be clearly delineated (A). B demonstrates measurements of the IOD, BOD, GD and LD. The eyeball is divided into lens and other structures, and was filled with different colors (C). D is the 3D visualization model of the eyeball and can dynamically demonstrate the eyeball. The bar in each figure represents 1 cm.

The largest transverse T2-weighted section through the eyeballs was selected (Fig 1A) to measure the linear interocular and binocular distances (IOD and BOD, respectively), globe diameter (GD) and lens diameter (LD) (Fig 1B) according to previous research [8, 10, 12]. Amira 4.1 was used for image segmentation and reconstruction. The eyeball was segmented into the lens and other structures, and was filled with different colors (Fig 1C). The 3D reconstruction models were automatically obtained after segmentation (Fig 1D), and then the globe and lens volumes (GV and LV, respectively) were obtained. For the two eyeballs, two anatomists simultaneously segmented all images twice to obtain a mean.

### Statistical analysis

Regression analysis was carried out between all the measurements and the GA, and the regression equation and correlation coefficient were obtained. All statistical work was performed via SPSS 17.0.

## Results

### Fetal ocular structures

T2-weighted MRI images can clearly delineate the fetal eyeball and the adjacent structures (Fig 1A). The lens is described as a circle with a low signal on T2-weighted MRI images, and the vitreous has a high signal.

Three layers can be observed in the eyeball wall. The inner and outer layers are described with a low signal on T2-weighted MRI images, and the middle layer has a high signal. The extraocular muscle (Fig 1A, white arrows) and optic nerve (Fig 1A, black arrows) are clearly and continuously delineated with a low signal.

### 3D reconstruction and quantitative measurements

The eyeball is divided into the lens and other structures, and filled in different colors. A 3D visualization model is obtained after reconstruction (Fig 1D) and can clearly show the entire morphology of the eyeball. The volume of the segmented structures was obtained automatically.

**Fig 2:**
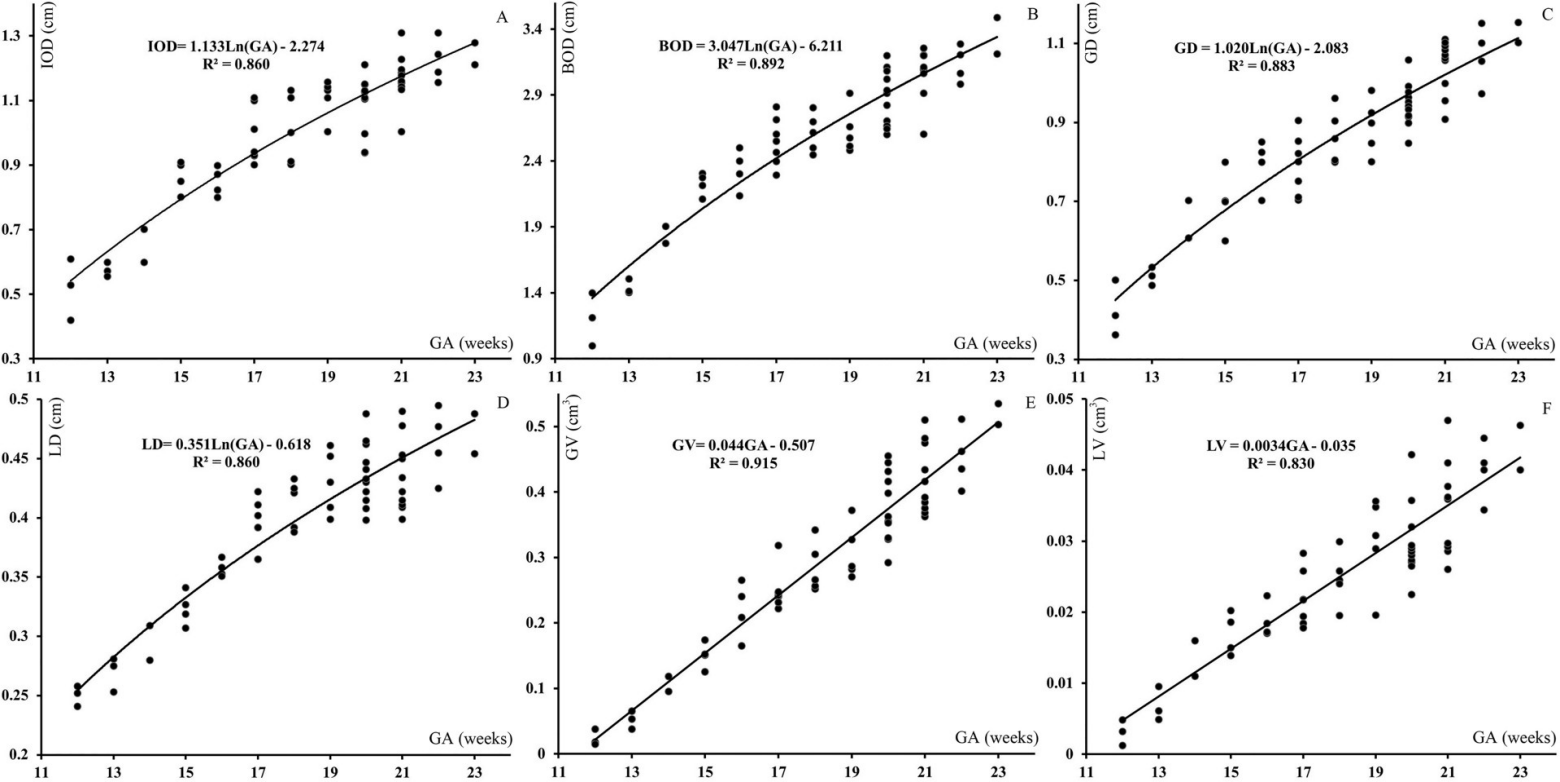
Quantitative measurements of fetal eyeballs in the second trimester and their relationship with GA. All the linear measurements (IOD, BOD, GD and LD) logarithmically increase with GA (A-D). However, the volumetric measurements (GV and LV) linearly increase with GA (E, F). There is a reasonably high correlation between all the measurements and GA.

All the linear measurements (IOD, BOD, GD and LD) logarithmically increase with GA (Fig 2A–2D). While, the volumetric measurements (GV and LV) linearly increase with GA (Fig 2E and 2F). There is a reasonably high correlation between all the measurements and GA.

The best fit regression equation and the regression coefficient are as follows: IOD = 1.133Ln(GA) -2.274, R^2^ = 0.860; BOD = 3.047Ln(GA) -6.211, R^2^ = 0.892; GD = 1.020Ln(GA) -2.083, R^2^ = 0.883; LD = 0.351Ln(GA) -0.618, R^2^ = 0.860; GV = 0.044GA -0.507, R^2^ = 0.915; LV = 0.0034GA -0.035, R^2^ = 0.830.

The 5% and 95% predicted confidence intervals of each measurement are listed in Table 2.

**Table 2:** The 5% and 95% confidence intervals for each measurement (cm or cm^3^) at different GA (weeks).

GA	Scalar values 5% and 95%					
	IOD	BOD	GD	LD	GV	LV
12	0.371/0.711	0.966/1.752	0.312/0.588	0.202/0.307	0/0.106	0/0.014
13	0.464/0.800	1.216/1.990	0.396/0.668	0.231/0.334	0/0.149	0/0.018
14	0.550/0.881	1.446/2.212	0.473/0.742	0.257/0.360	0.027/0.193	0.002/0.021
15	0.630/0.959	1.659/2.419	0.545/0.811	0.282/0.384	0.072/0.236	0.006/0.024
16	0.704/1.031	1.857/2.614	0.611/0.876	0.305/0.406	0.116/0.280	0.009/0.028
17	0.773/1.099	2.043/2.797	0.673/0.938	0.326/0.427	0.161/0.324	0.012/0.031
18	0.838/1.164	2.218/2.971	0.732/0.996	0.346/0.447	0.205/0.368	0.016/0.034
19	0.899/1.225	2.382/3.136	0.787/1.051	0.365/0.466	0.249/0.412	0.019/0.038
20	0.957/1.284	2.537/3.293	0.839/1.104	0.383/0.484	0.293/0.456	0.022/0.041
21	1.011/1.339	2.685/3.443	0.888/1.153	0.400/0.502	0.337/0.500	0.026/0.044
22	1.063/1.393	2.825/3.587	0.935/1.202	0.416/0.518	0.380/0.544	0.029/0.048
23	1.113/1.444	2.958/3.724	0.980/1.248	0.432/0.534	0.424/0.590	0.032/0.051

## Discussion

### Comparison with previous results

This study delineates fetal ocular structures in the second trimester of pregnancy using 7.0 T postmortem MRI. Normal quantitative measurements and a 3D visualization model of the fetal eyeball were obtained. We found that all the measurements increased regularly with GA and that these results may serve as a useful, precise reference of anatomy for in vivo fetal ocular development during this period.

Many growth charts for the fetal eyeball have been determined with US [7, 9]. Marwan et al. measured fetal eyeball volume of 14–40 weeks GA in 203 in vivo fetuses with 3D US [9]. Because both eyeball volumes could not be successfully measured in each fetus, the fetuses of 14–40 weeks GA were divided into several groups every two weeks [9]. Our results were consistent with theirs before 20 weeks GA. However, their US measurements were obviously twice the size of ours at 21–23 weeks GA. This difference may be explained by the fact that the human eyeball is not a typical ball, and its volume is not calculated based on one diameter of a section. A 3D US, limited by the image resolution, cannot clearly delineate the margin of the eyeball on each section. Therefore, the eyeball volume is defined as a typical globe based on one diameter of the largest section [9]. Thus, error arises and gradually increases with GA.

Most US eyeball measurements are obtained with reference to the bony landmarks of the medial and lateral orbital walls, which can be clearly delineated on the US [10]. Orbital measurements are ideally carried out on the axial plane, with equal access to and the largest possible diameters of both orbits. The BOD is measured between the two malar margins, and the IOD is defined as the linear distance between the two ethmoidal margins of the bony orbits. However, these bony landmarks are difficult to describe on fetal MRI scans, and standard sonographic growth charts cannot accurately or directly be applied to fetal MRI studies [10, 12].

At present, the imaging time of in vivo fetal MRI is shortened, and the image quality is greatly improved with the development of MRI techniques, especially the application of rapid acquisition sequences [12, 15]. Many researchers have described fetal ocular development with in vivo fetal MRI. Paquette et al. [10] obtained fetal ocular linear measurements of 127 in vivo fetuses at 17–39 weeks GA with 1.5 T MRI. In their results, there was only one fetus of 17 weeks GA, and fetuses of 18 and 19 weeks GA were missed with no measurements obtained during this period. During 17 and 20–23 weeks GA, our measurements of the GD and IOD were consistent with theirs. However, the LD was approximately 0.1 cm greater than that of Paquette’s. It is thought that this inconsistency is mainly caused by the differences in image resolution and slice thickness between in vivo and in vitro postmortem MRI measurements. The image resolution of the former is much lower than that of the latter, and the slice thickness is commonly 3–5 mm, which is much thicker than that used in our research (0.5 mm). The lens is a relatively small structure, and in vivo MRI can barely delineate it with just the largest diameter. However, postmortem MRI obtained with high magnetic strength is of high quality and is sufficient for eyeball delineation, segmentation, reconstruction and quantitative analysis [1–6]. Consequently, our measurements may be more accurate and convincing.

Velasco-Annis obtained normative ranges for fetal ocular biometrics of 114 fetuses between 19 and 38 weeks GA, using volumetric 3D images reconstructed from multiple sets of fast 2D slice acquisitions [12]. Their linear measurements for IOD, BOD and GD between 19 and 23 weeks GA were roughly consistent with ours, but the GV was slightly smaller because of the exclusion of the lens due to poor contrast with the vitreous. The 5% and 95% confidence intervals at 19–23 weeks GA in our study were smaller than those of Velasco-Annis’s. The reason may be that the measurements in our research were more similar for each subject due to the high resolution of postmortem MRI. As the author mentioned, the accuracy of all measurements obtained from the reconstructed 3D visualization model was limited by the effective resolution of the original 2D, in vivo, T2-weighted MRI, which was approximately 1 mm, and caused partial volume effect, which did not allow measuring of the scleral tissue’s thickness. Consequently, the measurements in Velasco-Annis’s study had an error margin of ±0.5 mm.

There are different increasing tendencies between the linear and volumetric measurements as function of GA. All the linear measurements logarithmically increase with GA. However, the volumetric measurements linearly increase with GA. It is thought that the volumetric measurements can better demonstrate the ocular developmental tendencies because they are obtained based on the 3D visualization model, reconstructed from all the MR images of the eyeball. The linear results and their increasing tendencies may be easily affected by an insufficient number of subjects and the small measured value itself. There is a reasonably high correlation between all the measurements and GA, which indicates that additional measurements would have been statistically unlikely to alter the shape of the defined models, as shown by the close fitting of the straight lines and logarithmical curves with the actual data points.

### The significance and advantages of studying fetal ocular development by means of postmortem MRI and a 3D visualization model

There are many fetal ocular developmental diseases in the clinical setting, and fetal eyeball malformations, such as anophthalmia, microphthalmia and other eyeball abnormalities, including coloboma and congenital cataract, can be found in these diseases [8, 10]. In anophthalmia, there is complete absence of the eyeball but presence of the ocular adnexa, such as the conjunctiva, eyelids and lacrimal apparatus. Anophthalmia can be divided into two groups. Eyeballs never form in primary anophthalmia, which is commonly associated with genetic syndromes or chromosomal abnormalities. Secondary anophthalmia is usually caused by an insult during fetal development in vivo, such as infection or a toxic, metabolic or vascular event. Microphthalmia can also be divided into two groups: unilateral or bilateral. It is defined as the macroscopic absence of the eyeball but with the presence of histologic eyeball remnants. Microphthalmia is estimated to occur in 1 in 5000 to 1 in 8300 live births and in 1 in 2400 pregnancies. Fetal developmental diseases of other systems can also be associated with eyeball malformations. They are skull/face/jaw/ear anomalies, limb abnormalities, abnormal nails, atrial septal defects, brain abnormalities and genital abnormalities [10]. It is thought that our quantitative measurements of normal fetal eyeballs could contribute to the detection of abnormalities of the eyeball.

There are several advantages of the 3D visualization model compared with traditional 2D imaging slices. It may be the most accurate method to study change in fetal organ morphology development and has been demonstrated in our previous research [1–3, 6]. First, it supplies 3D and 2D images of the fetal eyeball surface and inner structures without excising any tissue, thus precluding deformity produced by gravity or during excision. Second, arbitrary 2D slices can be obtained by postprocessing based on the 3D visualization model. Third, measurements of each segmented component can be exactly and easily obtained with the eyeball in situ, which may be more usable and accurate.

The fetal eyeball is a relatively small organ, and its presentation and measurements are easily affected by the image resolution. US and in vivo fetal MRI have difficulties clearly delineating and accurately measuring the fetal eyeball, especially for its anatomical boundaries and inner structures. The magnetic strength of fetal MRI in vivo is commonly 1.5 T at present, and the acquisition time is usually shorter than that of other common sequences. Fetal MRI in vivo is easily influenced by fetal movement, pulse of the maternal artery or by other structures [12, 15]. As a result, it has a relatively low image contrast compared with the more common in vitro MRI, which precludes the application of some postprocessing software for 3D reconstruction. The eyeball can only be observed in several sections of in vivo fetal MRI, and in most instances, only linear measurements can be obtained from one section. However, postmortem MRI, obtained with high magnetic strength, is of high image quality. Moreover, it is sufficient for segmentation, reconstruction, and the subsequent quantitative analysis because image acquisition of postmortem MRI can be carried out with increased scanning magnetic strength, lengthened imaging time and decreased thickness. It may be another ideal method to study fetal development.

### Limitations

There are several limitations of this study.

First, there are differences between in vivo and postmortem MRI. At present, in vivo fetal MRI is commonly performed at 1.5 T, which may limit the clinical application of the present results which were obtained with a higher field strength (7.0 T). Although our results may be more accurate, they cannot be routinely obtained with in vivo fetal MRI, especially the volume measurements (GV and LV). It is believed that the present results cannot be used directly in clinical settings; they may just provide certain beneficial information in evaluating and interpreting fetal eyeball development in vivo in the second trimester and may be considered a reference for MRI examination performed at lower field strengths (such as 1.5 T).

Second, all scanned fetal specimens had undergone formalin fixation, and the morphological differences between the formalin fixed brain and the in vivo samples should be considered. There is minor tissue degradation caused by formalin fixation, which affects the measurements and shape statistical results slightly in postmortem studies. However, previous studies have confirmed the clinical value of measurements obtained from postmortem tissues [1–6]. After fetal death, complex events take place in the histological environment, such as retinal detachment, which has been observed in most of our subjects. However, our study mainly concerns the total morphology of the fetal eyeball, and inner changes inside the eyeball can hardly affect the total measurement. Fetal eyeballs easily disintegrate or become malformed during collection and handling. Therefore, they were separately preserved in wide-mouthed bottles made according to their body size to avoid deformation once they were obtained and during transportation.

Third, the sample size of 60 fetuses in this study was relatively small, and there are no histological comparisons with high resolution MRI and no analysis of asymmetries and sexual dimorphisms in the parameters.

## Conclusions

Fetal ocular structures in the second trimester of pregnancy can be clearly document with 7.0 T postmortem MRI. The quantitative data presented here may be a valuable reference for the assessment of normal fetal eyeball development in clinical settings and as a supplement to anatomical investigations.

## Supporting information

S1 File The original measurements and charts for ocular biometrics. (XLS)
